# Improving Assessment, Diagnosis, and Management of Urinary Incontinence and Lower Urinary Tract Symptoms on Acute and Rehabilitation Wards That Admit Adult Patients: Protocol for a Before-and-After Implementation Study

**DOI:** 10.2196/22902

**Published:** 2021-02-04

**Authors:** Dianne Lesley Marsden, Kerry Boyle, Louise-Anne Jordan, Judith Anne Dunne, Jodi Shipp, Fiona Minett, Amanda Styles, Jaclyn Birnie, Sally Ormond, Kim Parrey, Amanda Buzio, Sandra Lever, Michelle Paul, Kelvin Hill, Michael R P Pollack, John Wiggers, Christopher Oldmeadow, Dominique Ann-Michele Cadilhac, Jed Duff, The I-SCAMP Project Team

**Affiliations:** 1 Hunter Stroke Service Hunter New England Local Health District Newcastle Australia; 2 Faculty of Health and Medicine University of Newcastle Newcastle Australia; 3 Brain and Mental Health Program Hunter Medical Research Institute Newcastle Australia; 4 Centre of Research Excellence in Stroke Rehabilitation and Brain Recovery Newcastle and Melbourne Australia; 5 Belmont Hospital Hunter New England Local Health District Newcastle Australia; 6 Rankin Park Centre Hunter New England Local Health District Newcastle Australia; 7 John Hunter Hospital Hunter New England Local Health District Newcastle Australia; 8 Manning Hospital and Wingham Hospital Hunter New England Local Health District Taree Australia; 9 Armidale Hospital Hunter New England Local Health District Armidale Australia; 10 Tamworth Hospital Hunter New England Local Health District Tamworth Australia; 11 Calvary Mater Newcastle Newcatle Australia; 12 Port Macquarie Hospital Mid North Coast Local Health District Port Macquarie Australia; 13 Coffs Harbour Hospital Mid North Coast Local Health District Coffs Harbour Australia; 14 Ryde Hospital Northern Sydney Local Health District Sydney Australia; 15 Susan Wakil School of Nursing and Midwifery The University of Sydney Sydney Australia; 16 Continence Service Hunter New England Local Health District Newcastle Australia; 17 Stroke Foundation Melbourne Australia; 18 Stroke Theme Florey Institute of Neuroscience and Mental Health University of Melbourne Melbourne Australia; 19 Health Research and Translation Hunter New England Local Health District Newcastle Australia; 20 Public Health Program Hunter Medical Research Institute Newcastle Australia; 21 Clinical Research Design & Statistics Hunter Medical Research Institute Newcastle Australia; 22 Stroke and Ageing Research, Department of Medicine School of Clinical Sciences Monash University Clayton Australia; 23 Centre for Healthcare Transformation Queensland University of Technology Brisbane Australia; 24 see Acknowledgments

**Keywords:** urinary incontinence, lower urinary tract symptoms, inpatient, practice-gap, practice improvement, protocol

## Abstract

**Background:**

Urinary incontinence (UI) and lower urinary tract symptoms (LUTS) are commonly experienced by adult patients in hospitals (inpatients). Although peak bodies recommend that health services have systems for optimal UI and LUTS care, they are often not delivered. For example, results from the 2017 Australian National Stroke Audit Acute Services indicated that of the one-third of acute stroke inpatients with UI, only 18% received a management plan. In the 2018 Australian National Stroke Audit Rehabilitation Services, half of the 41% of patients with UI received a management plan. There is little reporting of effective inpatient interventions to systematically deliver optimal UI/LUTS care.

**Objective:**

This study aims to determine whether our UI/LUTS practice-change package is feasible and effective for delivering optimal UI/LUTS care in an inpatient setting. The package includes our intervention that has been synthesized from the best-available evidence on UI/LUTS care and a theoretically informed implementation strategy targeting identified barriers and enablers. The package is targeted at clinicians working in the participating wards.

**Methods:**

This is a pragmatic, real-world, before- and after-implementation study conducted at 12 hospitals (15 wards: 7/15, 47% metropolitan, 8/15, 53% regional) in Australia. Data will be collected at 3 time points: before implementation (T_0_), immediately after the 6-month implementation period (T_1_), and again after a 6-month maintenance period (T_2_). We will undertake medical record audits to determine any change in the proportion of inpatients receiving optimal UI/LUTS care, including assessment, diagnosis, and management plans. Potential economic implications (cost and consequences) for hospitals implementing our intervention will be determined.

**Results:**

This study was approved by the Hunter New England Human Research Ethics Committee (HNEHREC Reference No. 18/10/17/4.02). Preimplementation data collection (T_0_) was completed in March 2020. As of November 2020, 87% (13/15) wards have completed implementation and are undertaking postimplementation data collection (T_1_).

**Conclusions:**

Our practice-change package is designed to reduce the current inpatient UI/LUTS evidence-based practice gap, such as those identified through national stroke audits. This study has been designed to provide clinicians, managers, and policy makers with the evidence needed to assess the potential benefit of further wide-scale implementation of our practice-change package.

**International Registered Report Identifier (IRRID):**

DERR1-10.2196/22902

## Introduction

### Background

Urinary incontinence (UI) and lower urinary tract symptoms (LUTS) are commonly experienced by adults admitted to hospitals, also referred to as inpatients, and contribute to the complexity and cost of providing care to these individuals. UI types include functional, neurogenic, stress, overflow, continuous, urgency, and mixed UI [1,2]. LUTS include acute and chronic urinary retention, frequency, urgency, and nocturia [1,2]. Surprisingly, data on the prevalence of UI and LUTS in adult inpatients are limited. However, these conditions have been reported to range from 10% to 45% of patients receiving acute and subacute care in hospitals [3-5]. The often-taboo subject of UI is significantly associated with poorer patient outcomes, including urinary tract or urinary catheter–associated infections [6,7], incontinence-associated pressure injury [8], falls [9], and pain associated with these conditions [6,7]. People with UI are twice as likely to experience depression and are more often socially isolated [6,7,10]. UI is associated with increased carer stress and is a main reason for carers feeling unable to continue in the carer role, leading to residential care admissions [11]. Although UI and LUTS are often complex and not always curable, with appropriate clinical care, symptoms can be managed and complications can be avoided.

International and Australian clinical practice guidelines provide recommendations for optimal care for UI and LUTS, based on the current, albeit limited research evidence [1,12-16]. Stroke is an example where UI and LUTS care has been included in condition-specific guideline recommendations [17-19]. Australian stroke guideline recommendations for optimal care are that all people poststroke are screened for continence issues and that those with symptoms receive an assessment, diagnosis, and a tailored inpatient and postdischarge management plan [17]. In the 2017 National Stroke Audit Acute Services, of the one-third of inpatients with UI, only 18% received a management plan [20]. In the 2018 National Stroke Audit Rehabilitation Services, of the 41% of inpatients who had UI, 52% had a documented management plan [21]. These results indicate an evidence-practice gap in current inpatient UI/LUTS care.

Although peak bodies recommend that health services have systems for optimal UI and LUTS care [1,15,17], there is little reporting of effective inpatient interventions to systematically deliver this care, as demonstrated in stroke care. In a recent Cochrane review, it was identified that there was limited evidence for the effectiveness of UI interventions poststroke [6]. The review included 20 trials (with 1338 participants, reporting 21 comparisons), with the authors reporting that the risk of bias was impossible to judge for many of the included studies because of poor reporting. The authors call for more robust multicenter trials.

As part of our formative quality improvement research [22], in 2009-2010, we translated high-level UI and LUTS guideline recommendations into an intervention that presents clear, concise, and explicit optimal inpatient care in a user-friendly format [23]. We collaborated with health service clinicians and managers from 3 rehabilitation services in the Hunter Region, Australia, to synthesize the best-available evidence into our Structured urinary Continence Assessment and Management Plan (SCAMP) intervention that was specifically designed for inpatients poststroke in metropolitan rehabilitation units [22]. Our SCAMP intervention consists of (1) a 4-page clinical decision support tool guiding comprehensive UI and LUTS assessment, diagnosis, and management; (2) the associated clinical practice guideline; and (3) supporting web-based education modules [22]. Stroke clinicians using the SCAMP intervention identified that it has the potential to be applicable across a range of hospitals in different health districts, for inpatients with a range of diagnoses including stroke, and across the phases of inpatient care.

### Aim

The aim of this study is to determine if the implementation of our SCAMP intervention is feasible and effective across this range of clinical scenarios.

### Research Questions

#### Primary

Does the implementation of our SCAMP intervention increase the proportion of inpatients with UI/ LUTS who have an individually tailored UI/ LUTS management plan?

#### Secondary

1. Does the implementation of our SCAMP intervention increase the proportion of:
  Inpatients with UI/LUTS who have an assessment and diagnosis of types of UI/LUTS?
  Inpatients with UI/LUTS and their caregivers who are involved in the development of the management plan?
  Clinicians who rate their knowledge, skills, and confidence in identifying the types of UI/LUTS and assessing, diagnosing, and managing UI/LUTS as good or very good?
2. Does the implementation of our SCAMP intervention reduce in-hospital complication rates associated with UI/LUTS or urinary catheterization?
3. Are any improvements in the above outcomes maintained at 12 months after implementation begins?
4. What are the potential economic implications (cost and consequences) for hospitals implementing our SCAMP intervention?

## Methods

### Design

**Figure 1:**
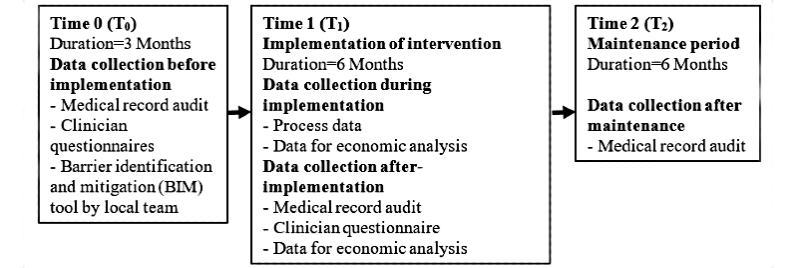
Study design and data collection timeframes.

This will be a pragmatic, real-world, before- and after-implementation study conducted at 12 hospitals. Data will be collected at 3 time points: before implementation (T_0_), immediately after the 6-month implementation period (T_1_), and again after a 6-month maintenance period (T_2_; Figure 1). Data will be collected from medical record audits and clinician questionnaires. An economic evaluation from the perspective of hospitals will be conducted. We will report our primary findings according to the Standards for Reporting Implementation Studies (StaRI) [24,25].

### Target Sites

Eleven hospitals in New South Wales (NSW) and 1 in Queensland, Australia, participated in our study. The hospitals are located in 4 health service districts. The hospitals were a convenience sample. Ten of the hospitals are located in 2 Local Health Districts that form part of the NSW Regional Health Partners, a Centre for Innovation in Regional Health (accredited by the National Health and Medical Research Council). Lead clinicians from the other 2 hospitals heard about our SCAMP intervention at conference presentations and approached the lead author about adopting the intervention.

Fifteen wards where patients after stroke are admitted are participating (Table 1). In Australia, people after stroke are cared for on wards that admit people with a range of conditions. This care may be provided in a stroke unit embedded in the ward or as part of the general ward population. This study was instigated by stroke clinicians who identified UI/LUTS inpatient care needed to be improved on their ward for people after stroke and potentially for other inpatient populations. To be eligible to participate, key ward clinicians and managers had to identify that UI/LUTS care was an issue for their ward and that they were willing to commit resources toward improving optimal UI and LUTS care by implementing the SCAMP intervention. The characteristics of each ward are outlined in Table 1. A total of 47% (7/15) of the wards are in 4 hospitals in 2 major cities, and the other 53% (8/15) wards are in 8 hospitals in inner regional locations [26]. The wards included 43% (7/15) rehabilitation wards, 27% (4/15) acute medical wards with an embedded stroke unit, 13% (2/15) wards with both acute and rehabilitation inpatients, 7% (1/15) rehabilitation ward with an embedded stroke unit, and 7% (1/15) medical ward. Before commencing the study, the investigators from each ward nominated the target adult inpatient populations from their ward to be included in the study of acute stroke, acute medicine, and/or rehabilitation for any condition, including stroke (Table 1). Clinician representatives from each site have been project team members from the outset (including authors KB, JAD, JS, FM, AS, JB, SO, AB, and SL).

**Table 1:** Characteristics of the participating wards.

Ward	Ward description	Hospital type and remoteness classification [26]	Previous use of SCAMP^a^ intervention	Patient populations included
1.	20-bedded rehabilitation ward	Principal referral or major city	Yes	Rehabilitation
2.	20-bedded rehabilitation ward	Principal referral or major city	No	Rehabilitation
3.	30-bedded rehabilitation ward: 22 rehabilitation or hospital overflow 8 neurological	Principal referral or major city	Yes	Acute medicine Acute stroke Rehabilitation
4.	12-bedded ward: 8 general medicine 4 acute stroke units	Public acute group A or major city	Yes	Acute medicine Acute stroke
5.	30-bedded ward: 26 general medicine 4 comprehensive stroke units	Public acute group B or major city	Yes	Acute medicine Stroke: acute and rehabilitation
6.	32-bedded ward: mixed medical and rehabilitation ward	Public acute group B or inner regional	Yes	Acute stroke Rehabilitation
7.	32-bedded rehabilitation ward	Public acute group B or major city	No	Rehabilitation
8.	28-bedded general medical ward	Public acute group B or major city	No	Acute medicine Acute stroke
9.	22-bedded rehabilitation ward	Public acute group A or inner regional	No	Rehabilitation
10.	28-bedded ward: 24 general medical 4 acute stroke unit	Public acute group A or inner regional	Yes	Acute stroke
11.	16-bedded rehabilitation hospital	Rehabilitation or inner regional	No	Rehabilitation
12.	28-bedded ward: 4 acute stroke units 8 medical assessment units 16 respiratory or cardiac units	Public acute group A or inner regional	No	Acute stroke
13.	24-bedded ward: 20 general rehabilitation 4 comprehensive stroke units	Public acute group A or inner regional	No	Stroke: acute and rehabilitation
14.	18-bedded hospital: 8 rehabilitation 10 general medical	Public acute group C or inner regional	No	Rehabilitation
15.	16-bedded rehabilitation ward	Public acute group A or inner regional	No	Rehabilitation

^a^SCAMP: Structured urinary Continence Assessment and Management Plan.

### Target Population

The population targeted by our practice-change package is clinicians (full time, part time, and casual) employed in each participating ward (including nurses, Nurse Unit managers, physiotherapists, occupational therapists, speech pathologists, social workers, and doctors). Participating clinicians are general medical, rehabilitation, or neuroscience clinicians who are not identified as continence or urology specialists. There are no exclusion criteria, as the study is a service improvement initiative, clinicians will not be consented to receive our practice-change package. The unit of analysis is hospital performance, based on patient-level data.

### Practice-Change Package (Study Intervention)

Our practice-change package is designed to support clinicians and health services to deliver guideline-recommended UI and LUTS care. It consists of our SCAMP intervention that we will implement using evidence-based implementation strategies.

#### Intervention

In 2018, we reviewed all 3 components of our SCAMP intervention with experts from stroke, continence, rehabilitation, and urology to ensure that they met the current best-evidence UI and LUTS care for the majority of adult inpatient populations. Our SCAMP intervention consists of the following:

- The 4-page SCAMP decision support tool, which has been approved by the Hunter New England Local Health District Forms Committee
- The associated Clinical Practice Guideline that includes UI Management Flowcharts modified from the International Continence Society flowcharts
- Eight web-based education modules and a local module on how to use the SCAMP decision support tool (a PowerPoint presentation with a voice-over). The web-based modules cover information on normal bladder function, why continence is an issue after stroke, and 6 of the common inpatient UI and LUTS types and are hosted on the Stroke Foundation website [27]

#### Implementation Strategies

To enhance the success of our SCAMP intervention, we will use evidence-based theoretical approaches for implementation [28,29]. As there is no one all-encompassing theory that guides implementation of a complex multicomponent intervention, we have chosen to use complementary approaches that align best with the various components of the study, including the project design, assessment of the barriers and enablers, systematic planning and development of implementation and sustainability processes, and the evaluation of the project [29]. The Knowledge to Action framework is a process framework that guides implementation [23]. The Theoretical Domains Framework is the determinant framework that will help us identify the constructs that may influence implementation (barriers and facilitators) [30,31]. The evaluation plan is informed by the RE-AIM (reach, effectiveness, adoption, implementation, maintenance) framework [32,33].

Implementation strategies were selected to overcome barriers identified by project team members with experience in implementation science and known barriers to clinicians implementing guideline recommendations identified in the literature [34,35]. Textbox 1 outlines the planned implementation strategies to support the practice change and how these strategies align with the Expert Recommendations for Implementing Change [36].

To identify ward-specific barriers, local teams will use the Barrier Identification and Mitigation tool [37]. Local teams will observe and ask clinicians about the SCAMP decision support tool and guideline and walk through the process to simulate real ward circumstances. From the data they collect during the identification phase, each team will summarize and prioritize barriers and then develop a local action plan. The practice-change package will be adapted by each site to suit their local context.

**Summary of planned implementation strategies.**

- Build a coalition. A coalition has been built that includes 15 wards across 12 hospitals, peak government and nongovernment bodies, and multiple universities.
- Work with educational institutions. Coalition members include institutions that provide tertiary and/or professional development education to the target groups.
- Develop academic partnerships and use data experts. Coalition members include academics from multiple institutions with expertise in implementation science, statistics, health economics, and data management.
- Centralize technical assistance. Sites will be supported by a centralized research team who will provide the evidence-based intervention (Structured urinary Continence Assessment and Management Plan; SCAMP); develop implementation resources in consultation with the team (including education materials and Implementation Training Workshops for site leaders); and evaluation resources (data collection tools, data storage, data analysis, and reporting).
- Access new funding. Sites will be supported to conduct the audits with small grants secured by the research team.
- Identify and prepare champions. Each site will have a local project lead and site champions who will drive the project locally. Leads will be senior clinicians, managers, or educators who have influence over local practice.
- Recruit, designate, and train for leadership. Site leaders will attend 2 training workshops that will include an overview what implementation research is and strategies for implementing evidence-based practice, overcoming barriers, generating sponsorship, communication, and using mixed methods for evaluation.
- Create a learning collaborative. A learning collaborative will be developed where sites learn from and share with each other to improve implementation.
- Develop resource-sharing agreements. Sites will share any implementation resources they develop with other members of the collaborative. This will be facilitated by a shared cloud–based repository.
- Organize clinician implementation team meetings and provide ongoing consultations. Project team members from each site will meet at 2 implementation workshops plus monthly teleconferences for education, consultation, and collaboration.
- Identify barriers and facilitators. Local sites will use the Behaviour Identification and Mitigation tool [37] to develop a local implementation plan.
- Tailor strategies and promote adaptability to meet local needs. Local implementation plans will tailor the implementation strategy and adapt the intervention to suit local needs.
- Distribute educational materials. Local sites will facilitate staff undertaking the education modules that inform the SCAMP decision support tool.
- Conduct educational meetings. Sites will conduct local education meetings to educate staff.
- Change record systems. The SCAMP decision support tool will be implemented at all sites. Paper or electronic versions will be used based on local needs.
- Audit and provide feedback. Before-implementation audit data will be fed back to each site.
- Remind clinicians. A poster display of different continence types and possible management solutions will be made available to all sites.

### Outcomes

Our primary outcome is the change (T_1_-T_0_) in the proportion of inpatients who have an individually tailored UI/LUTS management plan. This will be determined via a medical record audit.

Our secondary research outcomes are:

1. The change (T_1_-T_0_) in proportion of:
  - Inpatients with UI/LUTS who have an assessment and diagnosis of types of UI/LUTS, determined via a medical record audit
  - Inpatients with UI/LUTS and their carers who are involved in the development of the management plan, determined via a medical record audit
  - Clinicians who rate their knowledge, skills, and confidence in identifying the types of UI/LUTS and in assessing, diagnosing, and managing UI/LUTS as good or very good, determined via a clinician questionnaire
2. The change in in-hospital complication rates associated with UI/LUTS or urinary catheterization, determined via medical record audit (T_1_-T_0_)
3. The change in the aforementioned outcome measures at 12 months after implementation begins (T_2_-T_0_, T_2_-T_1_)
4. The potential economic implications for hospitals implementing our SCAMP intervention, determined using a cost-consequences analysis method

### Data Collection Procedures

Data will be collected at 3 time points (Figure 1): before implementation (T_0_), after a 6-month implementation period (T_1_), and after a 6-month maintenance period (T_2_). Before-implementation data will be used to tailor the intervention to each ward.

#### Medical Record Audit

Records of adults aged ≥18 years with the ward-nominated conditions who are discharged from each participating ward will be included. To reduce selection bias, we will include consecutive records of patients discharged from each ward for each month of the 3-month data collection period.

#### Screening

Records will be screened to determine if the patient had UI/LUTS, including an indwelling urinary catheter, during their stay on the participating ward. During screening, we will extract data, including demographic, characteristic information, continence status, and how the UI/LUTS status was determined. Patients will be excluded from the full medical record audit if they are determined to have had no UI/LUTS during admission to the participating ward—deemed palliative/at the end of life and died during their admission or were discharged with this care type. People deemed to be at the end of life will be excluded as their management goals for UI/LUTS are usually different from those receiving acute and rehabilitative treatment. Patients who have an unexpected death, for example, cardiac arrest, during admission will be included.

#### Audits

Medical record audits of patients with UI/LUTS, including those with an indwelling urinary catheter, will be performed for 15 records for each month or until all patients discharged during that month have been screened, whichever occurs first. The medical record audit tool is based on questions in the Australian Stroke Foundation National Audits [20,38,39] and the content of the SCAMP decision support tool. The medical record audit tool was designed by KB and DM, piloted and refined by the project team members, including KB, J Dunne, JS, FM, AS, JB, SO, AB, KP, and SL, who will be performing the audits. The authors then examined the tool for face validity. Medical record audits will be conducted at each hospital by the project team members from that hospital and other local clinicians with legitimate access to the medical records, as per local health service requirements for patient privacy and confidentiality. A web-based medical record audit data dictionary is available. Information regarding assessment, diagnosis, management, complications, level of disability, and the presence of comorbidities relevant to UI/LUTS will be extracted. Study data will be extracted into and managed using the REDCap electronic data capture tool [40], hosted on a secure server at the Hunter Medical Research Institute, NSW.

#### Clinician Questionnaire

Our web-based clinician questionnaire is aligned with 13 of the 14 domains of the Theoretical Domains Framework [31] of behavior change. The optimism domain was not included as we perceived an overlap with the emotions, beliefs about consequences, beliefs about capability, and goals domain questions. Selecting domains to include in a questionnaire is in keeping with other studies that have used the Theoretical Domains Framework [31,41].

The target population for our intervention will be approached via email or in person by their site project team members and invited to complete a deidentified web-based questionnaire. Local site project team members will not have access to individual participant results. Demographic data will include age range, profession, and years of clinical experience. The clinician questionnaire was designed by authors KB, JD, and DM.

#### Process Evaluation

Measures will be collected to assess the process and fidelity of the implementation of the intervention. Spot check audits will be conducted by site members of the research team and site champions to identify any local issues with completing the SCAMP decision support tool. This information will inform local strategies to address the identified issues. We will also record the attendance for ward education sessions and the project team implementation workshops, the monthly project team meetings, the number and availability of identified champions throughout, the number and types of resources generated and reminder activities conducted, the number of audit and feedback sessions conducted, and any local changes made to the SCAMP intervention [42].

#### Economic Evaluation

As there are multiple potential benefits and the cost impacts are unclear from the perspective of the hospitals, we will undertake an exploratory assessment of resource use and costs and present these as a cost-consequences analysis [43]. We will obtain data on the costs of implementing the package (including staff training) and the direct health costs attributable to eligible patients across each study time period for the management of UI/LUTS, and report any potential cost offsets related to the practice-change package. Costs will be valued based on the reference year 2019. Data sources will include screening log and patient-level data from the medical record audits, hospital finance department data, research literature, expert opinion, and project management or administrative data. Costs and outcomes (ie, proportion of inpatients with an individually tailored UI/LUTS management plan and complication rates associated with UI/LUTS or urinary catheterization) will be presented to provide context for the changes in costs relative to the benefits to aid in the future translational potential of this package. All individual health and nonhealth effects of the intervention, including various cost items, will be reported as summary measures, for example, point estimates with a measure of variability (*Data Analysis* section).

### Sample Size and Power Calculations

For the primary outcome, 15 consecutive medical record audits per site per month (ie, a pooled sample of 675 audits anticipated per data collection period) will provide >90% power to detect a 10% absolute increase (from before intervention) in the proportion of incontinent patients with a continence management plan (type 1 error rate of 5%). This calculation conservatively assumes that 20% of patients in acute and 50% of rehabilitation sites have a plan before intervention (based on the Australian Stroke Foundation National Audit results for included sites [20,39]).

### Data Analysis

The before-intervention group, after-intervention group, and maintenance period group results will be presented with descriptive statistics, including site, clinician, and medical record data for characteristics and demographics. No individual will be identifiable. All results will be presented as aggregated summary measures, with their variance depending on the distribution of the data (eg, mean and standard deviation, medians, and interquartile range). Groups will be compared with respect to change, from baseline (T_0_) to immediately postintervention period (T_1_) and from baseline to maintenance period (T_2_) using mixed effects logistic regression models, with a random intercept for site, and fixed effect for period. Results are presented as odds ratios with 95% CI and *P* values.

### Study Discontinuation

There are no criteria for study discontinuation as it is not anticipated that there are any events that would warrant discontinuation of this study. Any unforeseen adverse events will be reported to the Hunter New England Human Research Ethics Committee (the primary approval committee) and advice sought out regarding the required action. Any deviations from this original protocol will be reported in our study outcomes papers.

## Results

Preimplementation data collection (T_0_) was completed in March 2020. As of November 2020, 87% (13/15) wards have completed implementation and are undertaking postimplementation data collection (T_1_).

## Discussion

Our practice-change package is designed to reduce the current inpatient UI/LUTS care evidence-practice gap. We will contribute to the implementation research literature by demonstrating the potential impact of using a clinically applicable, evidence-based intervention that has been informed by the knowledge translation theory to optimize uptake in hospitals. We will describe the resources and costs associated with implementing the SCAMP intervention via a cost-consequences economic analysis. Our cost consequence analysis will provide an opportunity to pilot instruments used to collect economic data, such as resource use and clinical outcomes [43]. This analysis will be essential for establishing the benefit of scaling up the practice-change package. This study has been designed to provide clinicians, managers, and policy makers with the evidence needed to assess the potential benefit of further, wide-scale implementation of our practice-change package. We will report our findings according to the StaRI [24,25]. This will ensure that our practice-change package can be replicated in other clinical sites and in future research.

The results from this study will provide evidence to whether our UI/LUTS practice-change package is effective in supporting clinicians and health services deliver optimal care. To ensure that our practice-change package is evidence-based, clinically relevant, and applicable, it has been developed from the outset with our team of inpatient clinicians and managers, clinician researchers, and academics with experience in implementation science. To increase the generalizability and potential scalability of our practice-change package, we are testing it in a range of clinical scenarios and across the phases of inpatient care for people with a range of diagnoses, including stroke, admitted to metropolitan and regional hospitals in 4 health districts in 2 Australian states. It may also be applicable to other health conditions where providing optimal UI and LUTS care is challenging.

## Abbreviations

LUTS: lower urinary tract symptom
NSW: New South Wales
SCAMP: Structured urinary Continence Assessment and Management Plan
StaRI: Standards for Reporting Implementation Studies
T_0_: before implementation
T_1_: immediately after the 6-month implementation period
T_2_: after a 6-month maintenance period
UI: urinary incontinence
